# Green Tea and Epigallocatechin Gallate (EGCG) for the Management of Nonalcoholic Fatty Liver Diseases (NAFLD): Insights into the Role of Oxidative Stress and Antioxidant Mechanism

**DOI:** 10.3390/antiox10071076

**Published:** 2021-07-05

**Authors:** Guoyi Tang, Yu Xu, Cheng Zhang, Ning Wang, Huabin Li, Yibin Feng

**Affiliations:** 1 School of Chinese Medicine, Li Ka Shing Faculty of Medicine, The University of Hong Kong, 10 Sassoon Road, Pokfulam, Hong Kong, China; tanggy@connect.hku.hk (G.T.); xyzjh@hku.hk (Y.X.); zttc@connect.hku.hk (C.Z.); ckwang@hku.hk (N.W.); 2 School of Public Health, Sun Yat-sen University, Guangzhou 510080, China; lihuabin@mail.sysu.edu.cn

**Keywords:** green tea, epigallocatechin gallate, nonalcoholic fatty liver disease, nonalcoholic steatohepatitis, multiple parallel hits, oxidative stress, reactive oxygen species, redox homeostasis

## Abstract

Nonalcoholic fatty liver diseases (NAFLD) represent a set of liver disorders progressing from steatosis to steatohepatitis, fibrosis, cirrhosis, and hepatocellular carcinoma, which induce huge burden to human health. Many pathophysiological factors are considered to influence NAFLD in a parallel pattern, involving insulin resistance, oxidative stress, lipotoxicity, mitochondrial dysfunction, endoplasmic reticulum stress, inflammatory cascades, fibrogenic reaction, etc. However, the underlying mechanisms, including those that induce NAFLD development, have not been fully understood. Specifically, oxidative stress, mainly mediated by excessive accumulation of reactive oxygen species, has participated in the multiple NAFLD-related signaling by serving as an accelerator. Ameliorating oxidative stress and maintaining redox homeostasis may be a promising approach for the management of NAFLD. Green tea is one of the most important dietary resources of natural antioxidants, above which epigallocatechin gallate (EGCG) notably contributes to its antioxidative action. Accumulative evidence from randomized clinical trials, systematic reviews, and meta-analysis has revealed the beneficial functions of green tea and EGCG in preventing and managing NAFLD, with acceptable safety in the patients. Abundant animal and cellular studies have demonstrated that green tea and EGCG may protect against NAFLD initiation and development by alleviating oxidative stress and the related metabolism dysfunction, inflammation, fibrosis, and tumorigenesis. The targeted signaling pathways may include, but are not limited to, NRF2, AMPK, SIRT1, NF-κB, TLR4/MYD88, TGF-β/SMAD, and PI3K/Akt/FoxO1, etc. In this review, we thoroughly discuss the oxidative stress-related mechanisms involved in NAFLD development, as well as summarize the protective effects and underlying mechanisms of green tea and EGCG against NAFLD.

## 1. Introduction

Nonalcoholic fatty liver disease (NAFLD) is one of the most important public health issues induced without alcohol consumption to an unsafe extent or other clear cause [1]. The estimated morbidity of NAFLD is about 17–33% in the general population, while it dramatically reaches 75% in obese individuals, and even more in patients with type 2 diabetes mellitus (T2DM) [2,3]. NAFLD includes a wide spectrum of liver pathological conditions, ranging from simple steatosis to steatohepatitis (namely NASH), fibrosis, cirrhosis, and can eventually develop into hepatocellular carcinoma (HCC) [4]. Previously, the “Two Hits Hypothesis” was proposed to demonstrate the underlying mechanisms mediating the initiation and progression of NAFLD. Insulin resistance serves as the “first hit”, which leads to the disorder of triglycerides synthesis and transport and, as a result, free fatty acids (FFA) accumulate in the hepatocytes [5,6]. Subsequently, FFA deposition enhances the β-oxidation in mitochondria, raises the levels of cytochrome P450 4A (CYP4A), CYP2E1, and increases the formation of reactive oxygen species (ROS). The ROS-mediated oxidative stress is the “second hit” that triggers the onset of NAFLD [5,6]. The development of NASH requires the presence of the “second hit” [6]. Recently, in addition to the “Two Hits Hypothesis”, the “Multiple Parallel Hits Hypothesis” pointed out that not only insulin resistance and oxidative stress, but also lipotoxicity, adipokines secretion by adipocytes, endotoxins (lipopolysaccharide, LPS) released by gut microbiota, and endoplasmic reticulum (ER) stress, act in parallel to promote NAFLD progression from steatosis to NASH, fibrosis, and eventually end-stage liver diseases [7,8,9]. Furthermore, the environmental, nutritional, genetic, and epigenetic factors have also been documented in the pathophysiological basis of NAFLD [2]. Among these multiple factors, oxidative stress is deemed to play a very likely vital role as an initial response for the hepatic and extrahepatic injury [10,11,12]. Oxidative stress may promote hepatic lipid accumulation, infiltrated inflammation, interstitial fibrosis, and HCC during NAFLD [1,2,3,4]. Ameliorating oxidative stress induced by ROS and retaining redox homeostasis in the liver may serve as a favorable strategy for NAFLD prevention and management [10,13].

**Figure 1:**
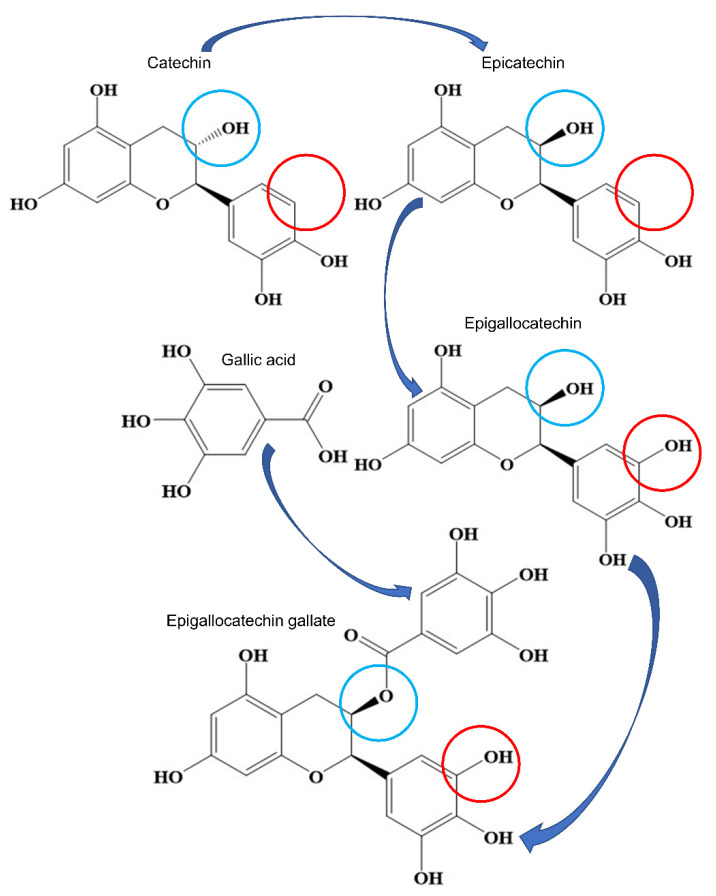
The relationship of epigallocatechin gallate (EGCG) and its derivates catechin, epicatechin, epigallocatechin, and gallic acid. It indicates that EGCG is the ester of epigallocatechin and gallic acid. The blue and red circles highlight the main structural differences of these compounds.

Green tea is usually produced using the bud and tender leaf of *Camellia sinensis* as raw material through simple stimming, rolling, and drying procedures, in which the natural antioxidants are seldom destroyed or converted as they can be in the fermentation process required for producing black and dark teas [14,15]. Green tea possesses potent antioxidant activity, owing to the plentiful tea catechins that have been identified as catechin, catechin gallate, epicatechin, epicatechin gallate, gallocatechin, gallocatechin gallate, epigallocatechin, and epigallocatechin gallate (EGCG) [14,15,16]. EGCG, a polyphenol formed by the ester of epigallocatechin and gallic acid (Figure 1), is the most abundant antioxidant catechin of green tea. Green tea and EGCG can be promising candidates for the prevention and management of cancer, obesity, diabetes mellitus, cardiovascular diseases, neural diseases, and liver diseases, owing to its potent antioxidant, anti-inflammatory, and anti-fibrogenic properties [17,18,19,20,21,22,23]. Specifically, consumption of green tea and EGCG has been reported with beneficial health functions against NAFLD, partially by metabolism regulation, antioxidation, anti-inflammation, and anti-fibrosis actions [24,25,26,27].

However, the underlying mechanisms involved in the pathophysiological progression of NAFLD, as well as the hepato-protective effects of green tea and EGCG against NAFLD, have not been fully elucidated. Thus, in this review, we firstly discuss the underlying mechanisms in NAFLD development, with emphasis on the relationship between oxidative stress and hepatic fat accumulation, inflammation, fibrosis, and carcinogenesis, and then summarize the hepato-protective effects of green tea and EGCG against NAFLD with highlights of related signaling pathways based on existing evidence. This review will provide comprehensive insights and prospective guidance for future research directions in this field.

## 2. Oxidative Stress in NAFLD Progression

In general, NAFLD progresses gradually from simple steatosis to steatohepatitis, fibrosis, cirrhosis, and HCC. The dysfunction of fatty acid β-oxidation, *de novo* lipogenesis, and lipid synthesis mediated by insulin resistance consequently leads to excessive fat deposition in the liver, namely simple fat accumulation (steatosis).

Following the stage of steatosis in the liver, NAFLD may progress in a more complicated status called steatohepatitis (NASH). Though the exact nature of subsequent hits after insulin resistance has not been completely elucidated, accumulative evidence has suggested the probable underlying mechanisms contributing to the progression from steatosis to NASH, in which oxidative stress-induced inflammatory cascades via adipokine secretion and cytokine activation is of such a critical importance that it appears to be responsible for inflammation initiation [11,28,29].

Fibrosis may progressively occur in NAFLD development, which serves as the major determinant of the long-term prognosis of NAFLD patients and the key indicator of increased mortality rate in NAFLD patients [30,31,32]. A systematic review and meta-analysis involving 4428 patients from 13 studies reported that fibrosis stage was associated with all-cause mortality and liver-related mortality, no matter whether potential confounding factors were adjusted or not [33]. Many factors and signaling pathways have been well documented in fibrogenesis and fibrosis in the liver under NAFLD condition. Oxidative stress has been reported to stimulate liver fibrosis directly or indirectly, though increasing the formation of proinflammatory cytokines and activating hepatic stellate cells (HSCs) [34,35,36].

Liver cancers mainly comprise HCC, intrahepatic cholangiocarcinoma, and hepatoblastoma, while HCC is the most common type in patients with chronic liver diseases and, coupled with NAFLD and NASH, is correlated to dramatically elevated liver-specific and overall mortality rates [37,38,39,40]. Almost all the processes of NAFLD, including fat deposition, oxidative injury, NASH, liver fibrosis, and cirrhosis, are correlated with the increased risk of HCC, and provide a fertile ground for the advancement of HCC [9,41,42,43,44]. Of note, however, the acute injury-inflammation–fibrosis–cirrhosis–HCC paradigm does not provide a causal link between these processes and HCC, but only an associative relationship; for instance, some patients may develop HCC without the occurrence of cirrhosis [45]. Additionally, some scientific studies have demonstrated the promoting role of oxidative stress in HCC carcinogenesis in NAFLD, regardless of any involvement or lack thereof of inflammation/fibrosis/cirrhosis [2,3,46].

Redox homeostasis is one of the most important balances in the human body with regard to inhibiting ROS over-production and scavenging excessive ROS by the antioxidant defense system, as both oxidant and antioxidant signaling are essential characteristics of redox homeostasis [47]. Disrupted redox homeostasis causes oxidative stress, which has been well documented in the literature to correlate with liver steatosis, NASH, fibrosis, and HCC, as discussed below.

### 2.1. Oxidative Stress and Liver Steatosis

According to the “Multiple Parallel Hits Hypothesis”, insulin resistance, induced by obesity, diabetes mellitus, metabolic syndrome, etc., is the trigger of NAFLD [8]. Insulin normally functions as a trigger of lipogenesis, an inhibitor of peripheral lipolysis via blocking hormone-sensitive lipase, and an indirect antagonist of mitochondrial FFA oxidation via increasing malonyl-CoA concentration [48,49]. Insulin resistance may mediate energetic metabolism dysfunction in the liver, especially the fatty acid β-oxidation, *de novo* lipogenesis, and very low-density lipoprotein–triglyceride/cholesterol synthesis which, along with excessive FFA delivered to the liver, results in the deposition of various lipid in hepatocytes, consequently liver steatosis, serving as the first hit for the initiation of NAFLD [7].

ROS, together with reactive nitrogen species (RNS), are the byproducts during intracellular energetic metabolism in various hepatic cells, mainly shown as free radicals (O_2_^•−^, HO^•^, NO^•^, NO_2_^•^, etc.) and nonradicals (H_2_O_2_, HOCl, ONOOH, etc.) [50]. ROS are intrinsic to cellular functioning and should exist at low and stationary levels in normal cells. However, ROS can cause irreversible damage to DNA, as they oxidize and modify some cellular components and prevent them from performing the original functions. For instance, the insulin signaling may be activated by millimolar concentrations of H_2_O_2_, which may stimulate metabolic functions of insulin by the tyrosine phosphorylation of the insulin receptor β-chain [51,52]. In addition, H_2_O_2_ may also modulate ATP binding, which is required for the receptor autophosphorylation process, indicating that the activity of insulin receptor kinase is oxidatively regulated [53]. However, the insulinomimetic effect of H_2_O_2_ is mainly regulated via inhibition of the catalytic activity of various protein and lipid phosphatases that are negative regulators and off-mechanisms of insulin signaling [54,55,56,57].

Excess of hepatic lipids could aggravate the ROS generation and accretion by affecting the physiological processes in several ROS generators, such as the mitochondrion, peroxisome, and ER [50]. In mitochondrion, the electron transport chain, α-ketoglutarate dehydrogenase, pyruvate dehydrogenase, glycerol phosphate dehydrogenase, and monoaminoxidase are considered as the major sites for ROS production [58,59,60,61,62]. In peroxisome, H_2_O_2_ is produced during fatty acid oxidation, which can be promoted by the acyl-CoA oxidase superfamily that transfers electrons directly to oxygen to generate H_2_O_2_ [63,64,65,66,67]. In microsome, fat oxidation also participates in the adaptive response following hepatic lipid deposition and redox imbalance, in which CYP4A and CYP2E1 are the main contributors of ROS formation and oxidative stress in NAFLD [68,69,70,71]. While in ER, the oxidative protein maturation with the break and form of disulfide bonds is driven by the oxidoreduction 1 α and protein disulfide isomerase, repetitively, and in each cycle, ROS is produced as a byproduct [72,73,74]. In addition to the above organelles, some enzymes, including nicotinamide adenine dinucleotide phosphate (NADPH) oxidase (NOX), xanthine oxidase, nitric oxide synthase (NOS), cyclooxygenases, and lipoxygenases in the cytosol and plasma membranes, may promote ROS formation during metabolism procedures as well [75,76].

Accumulation of ROS in the liver may induce oxidative stress, which in turn exacerbates fat accumulation in the liver, and eventually accelerates NAFLD development. This hit to the liver is even worse when cellular ROS shifts to other types with more severe toxicity [77]. In addition to prooxidant mechanism, the activity of endogenous antioxidant enzymes is usually inhibited in experimental model of NAFLD, including superoxide dismutase (SOD), catalase (CAT), and glutathione peroxidase (GPX) that are responsible for the metabolism of free radicals. The endogenous antioxidants mainly include the reduced glutathione, (GSH), nicotinamide adenine dinucleotide (NADH), and NADPH, with oxidized glutathione (GSSG), NAD^+^, and NADPH^+^ as their oxidized types, respectively. The intracellular redox status can be determined by the redox pairs GSH/GSSG, NADH/NAD^+^, and NADPH/NADP^+^ [12,78,79,80]. Under oxidative stress, these ratios are usually reduced. Taken GSH/GSSG as an example, it may decrease with the depletion of GSH and the increase in GSSG, accompanied with the impaired transportation of cytosolic GSH into the mitochondrial matrix where it exerts its functions [5].

As a result of the reductions in endogenous antioxidants, the fatty acid synthesis, cholesterol synthesis, and lipogenesis are suppressed, while the β-oxidation, tricarboxylic acid cycle, and mitochondrial function are elevated, which lead to higher generation of free radicals that induce oxidative stress in the liver [81,82,83,84,85]. Consequently, lipid peroxidation occurs, which is the chain of reactions of oxidative degradation of lipids, especially polyunsaturated fatty acids [73]. Lipid peroxidation is proceeded by a free radical chain reaction mechanism, followed by the production of thiobarbituric acid reactive substances (TBARS), malonaldehyde (MDA), and 4-hydroxynonenal (HNE) [86]. Excessive lipid peroxidation may activate the signaling pathways mediating ER stress, cell apoptosis, inflammation, and fibrosis.

This antioxidant defense system can be regulated by the nuclear factor erythroid 2-related factor 2 (NRF2) through antioxidant response elements (ARE) [87,88,89]. NRF2 can promote cell survival and adaptation against oxidative stress by regulating cytoprotective proteins, intracellular antioxidants, anti-inflammatory and detoxifying enzymes, and protect the liver against steatosis by restricting lipogenesis and by improving lipid β-oxidation [87]. Thus, NRF2 as a potential target enables the possibility to manage NAFLD by attenuating oxidative stress and by ameliorating metabolism dysfunction and fat accumulation in the liver.

### 2.2. Oxidative Stress and NASH

In the pathological progress of NASH, some adipokines play crucial roles as the proinflammatory factor. Adipokines, or adipocytokines, are unique cytokines secreted by adipose tissue, which possess multiple functions in many procedures, including energy metabolism, immunological response, and inflammatory cascades. Adiponectin is the most abundant adipose tissue-specific adipokines, which is mainly produced in mature adipocytes in white adipose tissue, and the levels of adiponectin expression and secretion are correspondingly increased during adipocyte differentiation [90,91]. Adiponectin has been demonstrated with anti-inflammatory, anti-atherogenic and anti-diabetic properties [91,92,93,94]. Besides adiponectin, leptin is another important adipokines secreted by adipose tissue. Leptin is able to inhibit anabolic pathways, activate catabolic pathways, inhibit appetite, stimulate energy expenditure, regulate pancreatic function, affect T cell generation and differentiation, and antagonize liver inflammation. Leptin-deficient (ob/ob) mice and leptin receptor-deficient (db/db) mice have been established as obese and diabetic animal models, showing severe obesity and diabetes with abnormal pituitary/adrenal hormone secretion, insulin resistance, insulinemia, hyperglycemia, hyperlipidemia, immune function impairment, and high risk of liver disease, in particular NAFLD [91,95,96]. In addition to the above adipokines, some cytokines also function in NASH development, serving in the adipokines/cytokines networks [97]. For instance, tumor necrosis factor-α (TNF-α), interleukin-1β (IL-β), and IL-6 can inhibit the function of adiponectin, which has anti-inflammatory properties, associating with a series of inflammatory cascades in the liver [11]. In addition, proinflammatory TNF-α, IL-1β IL-6, and endotoxin can boost the secretion of leptin, which has central and peripheral effects on the energy metabolism, immune system, and inflammatory cascades [11].

ROS accumulation, following with the peroxidation of diverse lipids, the damage of hepatocyte membranes, proteins, and DNA, and the release of inflammatory adipokines/cytokines, are the consequences of oxidative stress-mediated mechanisms in NASH. Accumulative evidence has pointed out the close link between oxidative stress and adiponectin and leptin. For instance, ROS can restrain adiponectin production in adipocytes, and administrating antioxidants to obese mice significantly increased the adiponectin production [98,99]. While leptin may upregulate ROS formation that induces oxidative stress and promotes inflammation, activated NRF2 signaling can improve leptin resistance and subsequently alleviate oxidative stress-related pathological lesions, including inflammation [100]. In addition, ROS in company with the products of lipid peroxidation can increase the release of several cytokines, such as TNF-α, IL-1β, and IL-6 which play a vital role in inflammation, as well as induce the expression of TNF receptor-1 [28]. ROS also triggers the activation of nuclear factor-κB (NF-κB), a redox-sensitive transcription factor, which consequently promotes TNF-α expression. All these alterations may result in the occurrence and evolution of liver inflammation in NAFLD [29]. Meanwhile, it has been reported that NRF2 activation ameliorates liver inflammation by inhibiting the NF-kB pathway, and NF-kB may negatively modulate NRF2 transcription and lead to the deterioration of oxidative stress, partially explaining the interaction between oxidative stress and inflammation [87].

### 2.3. Oxidative Stress and Liver Fibrosis

ROS and aldehydes, a secondary product of the oxidation reaction, can activate HSCs, which shift into myofibroblasts in terms of phenotype with collagen-producing properties, subsequently leading to liver fibrosis via producing extracellular matrix proteins such as collagen I (COL I), COL III, COL IV, fibronectin, and α-smooth muscle actin (α-SMA) [34]. In addition, activated HSCs also excrete cellular factors that decrease the degradation of extracellular matrix, which further damages the balance between synthesis and degradation of these matrix constituents, and promotes its deposition. ROS can also activate NF-κB, inducing the increased expression of transforming growth factor-β (TGF-β), which has been commonly recognized as a key mediator in tissue fibrosis [7]. In patients with NASH, TGF-β can be produced by Kupffer cells (liver macrophages), which also activates HSCs and boosts liver fibrosis, with phagocytosis of apoptotic bodies as mediators of HSCs’ activation and fibrogenesis advancement [35,36]. In this context, the Kupffer cells and HSCs are deemed to play a crucial role as the decisive cell types in the pathogenesis of liver fibrosis, and the stimulation by ROS overproduction and lipid peroxidation has been regarded as the most important factor to induce liver fibrosis. Natural killer (NK) cells, significantly increased due to the up-expression of cytokines including IL-12, IL-18, and interferon γ (IFN-γ) in livers following oxidative stress and inflammatory response, are also associated with liver fibrosis progression [46]. In the early stage, NK cells exert anti-fibrotic effects by regulating IFN-γ and inducing HSCs apoptosis; in the late stage, NK cells function by increasing ECM deposition, subsequently leading to liver fibrosis [101,102].

Besides the above cells, portal fibroblasts and bone marrow-derived myofibroblasts may be recruited to the liver. These cells exert profibrogenic properties after activation by TGF-β and other inflammatory factors. Mastocytes originate from hematopoietic progenitor cells in the portal areas and fibrous septa. Recent studies also reported that mastocytes are involved in the pathogenesis of liver fibrosis in patients with NAFLD, as mastocytes contain many cytoplasmic stimulators and cytokines such as TGF-β. In addition, mastocyte degranulation may influence the extracellular environment via the induction of inflammation and the attraction of other inflammatory cells resulted from oxidative stress, ER stress, and other acute damages, subsequently leading to fibrogenesis in the liver [103].

### 2.4. Oxidative Stress and HCC

On the one hand, excessive oxidative stress due to the dysfunction of lipid metabolism, the formation of lipotoxic metabolites, and the release of ROS, may have direct revulsive effects on hepatic carcinogenesis [2,3]. The mechanisms involved in oxidative stress-mediated carcinogenesis comprise the modulation in cell-growth/survival and cancer-relevant signaling pathways (e.g., signal transducer and activator of transcription 1/STAT 1, STAT 3, TNF-α, NF-κB, IL-1, and IFN-γ) and the accumulation of oncogenic mutations (e.g., p53, Wnt, Notch, cIAP1, and Yap) via free radicals-induced DNA damage, DNA repairment inhibition, and telomere shortening, resulting in the alterations of both genetics and genomics [104,105,106,107,108,109,110].

On the other hand, oxidative stress may indirectly influence HCC initiation, growth, angiogenesis, and metastasis by changing the tumor microenvironment, which consists of the encircling blood vessels, infiltrated immune cells, fibroblasts, signaling molecules, and the extracellular matrix [111,112,113]. Oxidative stress also promotes the development of chronic inflammation, fibrosis, and cirrhosis, by stimulating the excretion of cytokines, which are key features of a permissive HCC microenvironment [114,115]. In addition, inhibition on immunosurveillance and the activation of hepatic progenitor cells and stellate cells by oxidative stress also contribute to the development of HCC [116,117].

**Figure 2:**
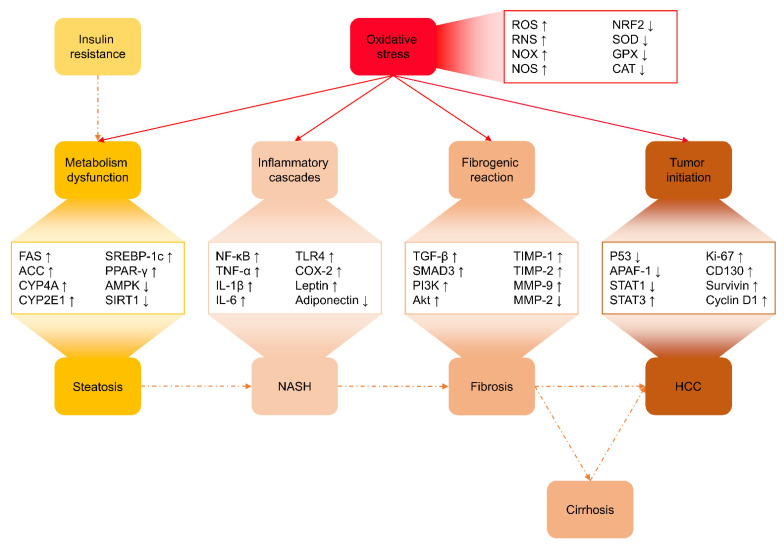
The crucial role of oxidative stress as a mediator, stimulator, and accelerator of other pathogenesis, including metabolism dysfunction, inflammatory cascades, fibrogenic reaction, and tumor initiation, in NAFLD progression. Of note, some of the molecules shown here may not have been mentioned in this section, but the next one. Abbreviations: ROS, reactive oxygen species; RNS, reactive nitrogen species; NOX, nicotinamide adenine dinucleotide phosphate (NADPH) oxidase; NOS, nitric oxide synthase; NRF2, nuclear factor erythroid 2-related factor 2; SOD, superoxide dismutase; GPX, glutathione peroxidase; CAT, catalase; FAS, fatty acid synthase; ACC, acetyl-CoA carboxylase; CYP, cytochrome P450; SREBP-1c, sterol element-binding protein 1c; PPAR-γ, peroxisome proliferator-activated receptor γ; AMPK, AMP-activated protein kinase; SIRT1, sirtuin 1; NF-κB, nuclear factor-κB; TNF-α, tumor necrosis factor-α; IL, interleukin; TLR4, Toll-like receptor-4; COX-2, cyclooxygenase-2; TGF-β, transforming growth factor-β; SMAD3, small mothers against decapentaplegic 3; PI3K, phosphoinositide 3-kinase; Akt, protein kinase C; TIMP, tissue inhibitor of metalloproteinases; MMP, matrix metalloproteinase; APAF-1, apoptotic protease activating factor 1; STAT, signal transducer and activator of transcription; NASH, non-alcoholic steatohepatitis; and HCC, hepatocellular carcinoma.

Taken together, oxidative damage to mitochondria alters mitochondrial respiratory chain polypeptides and mitochondrial DNA to partially block the flow of electrons in the respiratory chain and increase mitochondrial ROS formation, leading to a vicious cycle of damage amplification. ROS triggers lipid peroxidation, release of inflammatory cytokines, and cell death. Both biologically active lipid peroxidation products and cytokines act together to promote the diverse hepatic lesions of NAFLD by inducing hepatic inflammation and fibrosis that lead eventually to end-stage liver disease such as cirrhosis and even HCC. In the advancement of NAFLD, from simple steatosis, to NASH, fibrosis, cirrhosis, and HCC, oxidative stress plays a crucial role by acting as a mediator, stimulator, and accelerator (Figure 2).

## 3. Protective Effect of Green Tea and EGCG against NAFLD in Animal Study

As discussed above, oxidative stress has been regarded as a central mechanism in the development of NAFLD, contributing to steatosis, steatohepatitis, fibrosis, cirrhosis, and HCC. NAFLD is usually aggravated by the impaired antioxidant pathways, showing depletion of antioxidants such as GSH, vitamin C, and vitamin E, with low antioxidant enzyme activity and insufficient antioxidant defense [118,119]. Excessive ROS production results in the damage of mitochondrial DNA, the upregulation of uncoupling protein 2, the reduction in respiratory chain complex activity, and the impairment of mitochondrial β-oxidation, all of which leads to mitochondrial dysfunction that promotes the development of NASH and even advanced NAFLD [120].

In an ob/ob mice NASH model, supplementation with green tea extracts (0.5% and 1% in diet, 6 weeks) showed inhibitive effects on liver steatosis, NASH, and damaged liver function, which may be associated with the lowered hepatic NADPH activity, myeloperoxidase (MPO) expression, and ROS generation, along with reduced lipid peroxidation [118]. In an HFD-induced NASH model in Wistar rats, EGCG (1 g/L in drinking water, 6 weeks) alleviated the HFD-induced steatosis, steatohepatitis, ballooning degeneration, and necrosis in the liver, with improvements in the blood levels of ALT, triglyceride, insulin, and glucose, which was realized by improving oxidative stress, lipid peroxidation, and lipid metabolism, as revealed by the altered levels of GSH, MDA, and CYP2E1 [119]. In a NASH model induced in male Wistar rats by choline-deficient HFD plus repeated intraperitoneal injections of nitrite, administration with fermented green tea extracts (100 and 300 mg/kg BW, daily, 6 weeks) decreased serum AST and alkaline phosphatase (ALP) levels and improved liver steatosis and fibrosis, which may result from the prevention of lipid peroxidation, mitochondrial ROS production, and radical scavenging activity [120].

NRF2 is a key factor to limit oxidative stress by transcriptional activities, regulating xenobiotic metabolism and antioxidant defense system via ARE. NRF2 can also alleviate NASH through multiple mechanisms, including regulating the expressions of genes regarding pro-inflammatory response and lipid metabolism (e.g., lipogenic and cholesterogenic pathways), and mitigating oxidative stress during NASH by improving redox status concerning glutathione biosynthesis and the expressions of antioxidant enzymes (e.g., NADPH:quinone oxidoreductase 1/NQO1, SOD, and GPX) [121]. It has been reported that supplementation with green tea extract (2% in diet, 8 weeks) could increase NRF2 and NQO1 mRNA expressions, and reduce hepatic steatosis, lipid uptake, lipogenic gene expression, lipid peroxidation, and NF-κB-dependent NASH; while in NRF2-null mice, green tea extract lowered NF-κB phosphorylation and TNF-α and monocyte chemoattractant protein-1 (MCP-1) mRNA in a NRF2-independent manner, without improving the hepatic antioxidants α-tocopherol, ascorbic acid, and uric acid [121]. Taken together, it should be remarked that NRF2 deficiency may exacerbate NASH, whereas green tea extract may exert anti-inflammatory and hypolipidemic activities in both NRF2-dependent and NRF2-independent manner.

Of note, an extremely high dose of EGCG intake may potentially induce some side-effects, thus a relatively low or safe dose of EGCG supplementation to humans is warranted. The efficacy and safety of single administration of EGCG (160 mg/kg BW, the maximum safe dose had touched the contentious edge), EGCG (40 mg/kg BW, similar to the daily intake), caffeine (20 mg/kg BW), and the coadministration of EGCG (40 mg/kg BW) and caffeine (20 mg/kg BW) against NAFLD have been evaluated in obese rats. The results recommended that the coadministration of EGCG and caffeine exerted more outstanding effects on NAFLD, as revealed by the reduced body weight gain, white adipose tissue weight, decreased energy intake, and NAFLD-related liver injury. The underlying mechanisms may involve the improvements in serum lipid profile, oxidative stress, and adipose-derived and inflammatory cytokines, which is similar to the effect of high dose EGCG, but without safety anxiety [122]. It suggested that the combination intake of EGCG with other functional phytochemicals may be superior to high dose EGCG alone to treat NAFLD, with regard to the augmented efficacy and reduced side-effects. We should take into consideration the safe dose when applying EGCG to NAFLD management.

Based on the above demonstrations, it can be summarized that green tea and EGCG possess remarkable inhibitive effect against oxidative stress, mainly by NRF2 signaling pathways. These actions enable green tea and EGCG with the potential to alleviate NAFLD-related pathogenesis that are directly or indirectly associated with oxidative stress as described in the previous section, which are further discussed below.

### 3.1. Improvement of Liver Steatosis

In the early stage of NAFLD, lipid metabolism dysfunction occurs in the liver. Insulin resistance results in the activation of the lipolytic signaling pathway in the adipose tissue and augments FFA uptake into the liver, which increases the secretion of very low-density lipoproteins (VLDLs) and apolipoprotein B-100 into the circulation, further leading to the elevation of hepatic glucose production by gluconeogenesis and the activation of the *de novo* lipogenesis pathway in the liver [123]. Due to insufficient VLDL-TG synthesis, FFA overload, followed by the increase in triacylglycerol (TG) level, results in TG accumulation in hepatocytes, initiating fatty liver [123]. Green tea and EGCG have been shown to improve insulin resistance, lipid absorption, lipid metabolism, and hepatic lipid accumulation, thus exerting beneficial effects against NAFLD, among these actions the upregulations in AMP-activated protein kinase (AMPK) and sirtuin 1 (SIRT1) have been highlighted [124,125,126,127,128].

AMPK plays a crucial role in regulating *de novo* lipogenesis in liver, and its inhibition on hepatic lipogenesis has been documented as a potential therapeutic strategy for the prevention of NAFLD initiation and progression [123]. AMPK can be activated after phosphorylation, and liver kinase B1 (LKB1) phosphorylation may be required for the phosphorylation of AMPK [129]. Activated AMPK then possesses the ability to modulate lipogenesis through the phosphorylation and inactivation of acetyl-CoA carboxylase (ACC) that converts acetyl-CoA to malonyl-CoA, leading to the reduction in substrate flow for fatty acid synthase (FAS) and activity of FAS [130]. Moreover, AMPKα activation may decrease nuclear levels of sterol element-binding protein 1c (SREBP-1c) and carbohydrate response element-binding protein (ChREBP), indicating that AMPKα is a negative regulator of SREBP-1c and ChREBP [131].

SIRT1, a NAD^+^-dependent deacetylase that plays a key role in the regulation of lipid and glucose homeostasis, regulation of mitochondrial biogenesis, and control of insulin sensitivity and oxidative stress, may also serve as a potential therapeutic target for treating NAFLD [132]. The expression of SIRT1 was significantly reduced in a rat model of NAFLD induced via high-fat diet, while SIRT1 up-expression was found to have protective effect against NAFLD in mice [129]. SIRT1 functions, in whole or in part, by activating AMPK via inducing deacetylation of LKB1 under adverse situations that may lead to intracellular stress, including hypoxia, insulin resistance, and oxidative stress [129]. As for the upstream signaling, it was found that escalated levels of adiponectin and its receptors positively correlate with the activation of SIRT1, in which adiponectin acts as a post-transcriptional regulator that influences the protein, but not mRNA expression level of SIRT1 [123].

In high fat diet (HFD)-fed Swiss mice, supplement with green tea extract (50mg/kg BW, daily, 16 weeks) remarkably prevented weight gain and fatty liver, accompanied with reduced serum FFA level, and increased hepatic VLDL-TG secretion, by increasing expressions of SIRT1, p-AMPK, p-LKB1, and adiponectin receptor-2, while decreasing the expressions of ACC, FAS, SREBP-1c, and ChREBP [123]. In C57BL/6 mice fed with HFD, green tea extract supplementation (30, 60, and 120 mg/kg BW, daily, 12 weeks) was observed to reduce body weight gain, prevent hepatic fat accumulation, decrease hypertriglyceridemia and hyperglycemia, and improve insulin resistance, which might involve the upregulation of SIRT1, and AMPK followed with the downregulation of enzymes related to *de novo* lipogenesis [129]. In a model of NAFLD induced by HFD in genetically obese Zucker fatty rats, green tea polyphenol treatment (200 mg/kg BW, daily, 8 weeks) significantly suppressed hepatic triglyceride (TG) accumulation, and decreased cytoplasmic lipid droplet, which was associated with the significantly increased expression of AMPKα, reduced activation of ACC, and decreased expression of SREBP-1c following with diminished hepatic lipogenesis and triglycerides out flux from liver [130].

**Figure 3:**
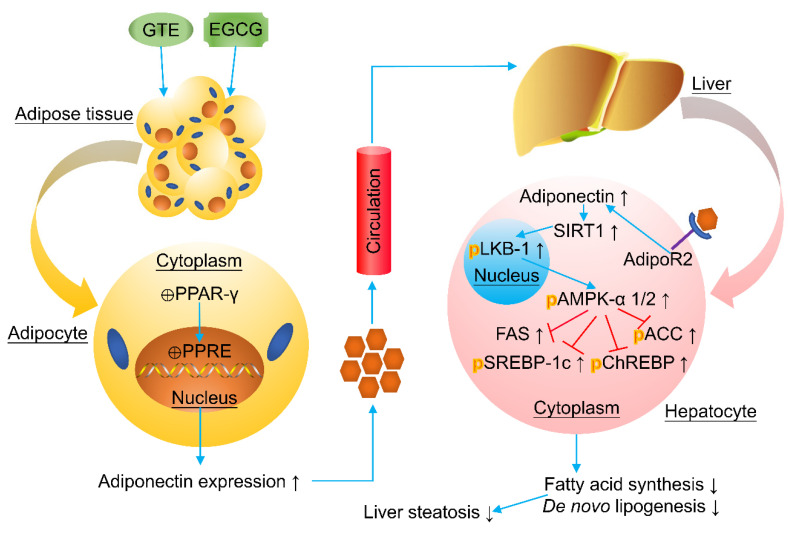
Green tea extract (GTE) and epigallocatechin gallate (EGCG) may ameliorate liver steatosis in NAFLD by improving lipid metabolism via targeting SIRT1 and AMPK signaling pathways. Abbreviations: PPAR-γ, peroxisome proliferator-activated receptor γ; PPRE, PPAR-responsive element; AdipoR2, adiponectin receptor 2; SIRT1, sirtuin 1; LKB1, liver kinase B1; AMPK, AMP-activated protein kinase; FAS, fatty acid synthase; ACC, acetyl-CoA carboxylase; SREBP-1c, sterol element-binding protein 1c; and ChREBP, carbohydrate response element-binding protein.

In addition to the regulation of AMPK and SIRT1 signaling pathways, the effects of green tea and EGCG against fatty liver may also be attributed to modulations in the protein kinase C (PKC/Akt) pathway and microRNAs [131,133]. In senescence-accelerated mice prone 8 (SAMP8), EGCG supplementation (3.2 g EGCG/kg chow diet) for 12 weeks improves insulin resistance by enhancing AMPKα activity, restoring Akt activity, recovering GLUT4 protein expression, and augmenting mitochondrial biogenesis in the skeletal muscle, and alleviates hepatic fat deposition by downregulating mTOR and SREBP-1c-mediated lipid biosynthesis via suppressing the positive regulator Akt and activating the negative regulator AMPKα in the liver [131]. In another study, it was also reported that the beneficial effect of green tea against fat accumulation in NAFLD could be attributed to the downregulation of hepatic miR-34a, with increases in its mRNA targets Sirt1, Pparα, and Insig2, as well as the upregulation of hepatic miR-194, with decreases in its target genes Hmgcs/Apoa5 [133]. Figure 3 summarizes the underlying mechanisms involved in the beneficial effect of green tea and EGCG against liver steatosis [123,129,130,131].

### 3.2. Amelioration of NASH

NASH is a clinicopathological entity characterized by chronic hepatic inflammation accompanied with steatosis in the liver. Once developed with NASH, the progression to end-stage liver disease, including fibrosis, cirrhosis, and HCC, may be accelerated in as little as a decade, thus treatment of NASH is of great importance to patients with NAFLD. Oxidative stress and/or proinflammatory insults are crucial for NASH development.

NF-κB, a transcription factor that critically modulates inflammatory gene expression, is involved in NASH progression. In NAFLD, NF-κB can be activated in a redox-dependent manner by oxidative stress, and blockage of NF-κB activation inhibits the release of proinflammatory cytokines, including TNF-α, IL-1β, IL-6, and MCP-1 [134,135,136]. MCP-1, a potent chemoattractant for monocytes, basophils, and memory T cells, can be induced by NF-κB and TNF-α, and may contribute to the progression of inflammatory diseases. Alleviating NASH by targeting NF-κB is a promising strategy to prevent the progression of NAFLD. In a study with nuclear SREBP-1c transgenic mice, EGCG (0.05% and 0.1% in drinking water, 12 weeks) was shown to reduce insulin resistance, oxidative stress, liver inflammation, and related liver injury, owing to the decreased expressions of pNF-κB, pAkt, and pIKK-β (inhibitor of nuclear factor kappa-B kinase) [134]. In another study, green tea extract (1% and 2% in diet, 8 weeks) protected against HFD-induced NASH in Wistar rats, and the mechanisms may involve the improved glutathione status associated with the inhibition of NF-κB-mediated inflammatory responses in liver and adipose [135].

Toll-like receptor-4 (TLR4)-mediated NF-κB activation as extracellular signaling, along with ROS-mediated intracellular signaling, is also a prominent process to induce NASH [137]. Ligands for TLR4 include gut-derived endotoxins (such as LPS) and saturated fatty acids (SFA), which usually increase in rodent models of NASH. Upon ligand binding, TLR4 functions applying adaptor myeloid differentiation primary response 88 (MYD88). Reducing the availability TLR4 ligands and/or inhibiting the hepatic TLR4 signaling may serve as a good strategy to block NF-κB-mediated inflammation in NASH. Dietary consumption of green tea extract could reduce NASH degree by lessening proinflammatory signaling through TLR4 and TNF receptor-1, which in turn augment NF-κB activation and promote NASH formation [137]. In wild-type and loss-of-function TLR4-mutant mice fed with HFD, green tea extract (2% in diet, 8 weeks) protected against inflammation in NASH, which was likely achieved by blocking the translocation of gut-derived endotoxin and TLR4/MYD88/NFκB activation, followed with lowered phosphorylation of the NF-κB p65 subunit and gene expressions of pro-inflammatory factors (TNF-α, MCP-1, MPO, and iNOS/inducible nitric oxide synthase) [138].

Oxidative stress-induced lipid peroxidation also increases the level of proinflammatory molecule cyclooxygenase-2 (COX-2), which is transcriptionally regulated by NF-κB (like TNF-α and iNOS) and catalyzes prostaglandin E2 (PGE2) synthesis [139]. Through a positive feedback system facilitated by NF-κB, PGE2 can increase its own biosynthesis by upregulating COX-2 and other NF-κB-dependent inflammatory cytokines. In turn, COX-2 can increase oxidative stress by enhancing ROS generation, and by aggravating PGE2-dependent inflammation in NASH. In HFD-fed Wistar rats, green tea extract supplementation (1% and 2% in diet, 8 weeks) restored the increased hepatic COX-2 protein and activity, as well as the PGE2 concentration and total hepatic n-6 and n-3 polyunsaturated fatty acid, without affecting the n-6/n-3 ratio, indicating green tea extract can attenuate lipid peroxidation and PGE2-mediated inflammation in liver via reduction in COX-2 activity, independent of arachidonic acid availability [139]. Of note, nitric oxide produced from iNOS is one of the most important RNS, which comprise another aspect of oxidative stress besides ROS. RNS can induce the expression of COX-2 and enhance the activity of COX-2, which increases the release of PGE2, further provoking inflammation in the liver. The protective role of green tea against NASH via regulating COX-2/PGE2 signaling pathway may also associate with the modulation on iNOS gene expression and nitric oxide production.

### 3.3. Attenuation of Liver Fibrosis

Following steatosis and steatohepatitis, NAFLD develops into the stage of liver fibrosis characterized with scar tissue around the liver and nearby blood vessels, which accelerate cirrhosis and HCC [140]. Hepatic stellate cells (HSCs) are crucial in liver fibrosis, as they can synthesize and excrete fibrogenic proteins after activation [136]. The TGF-β pathway has been well documented in the pathogenesis of liver fibrosis in NAFLD, in which TGF-β serves as a pleiotropic cytokine that regulates the SMAD (small mothers against decapentaplegic) signaling, and TGF-β/SMAD is a typical pathway that stimulates HSCs activation and extracellular matrix protein generation and deposition [136,140]. Besides the TGF/SMAD pathway, the phosphoinositide 3-kinase/protein kinase C/forkhead box protein O1 (PI3K/Akt/FoxO1) pathway is also a key modulator for liver fibrosis in NAFLD [140].

In methionine- and choline-deficient diet-induced NASH in male C57BL/6 mice, treatment with EGCG (25, 50, and 100 mg/kg BW, i.p., daily, 4 weeks) inhibited the mRNA expressions of TGF-β, COL I-α1, tissue inhibitor of metalloproteinases 1/TIMP-1, and α- SMA, as well as the phosphorylation of SAMD2 and SMAD3 in the liver and HSCs (LX-2 cells), suggesting that EGCG could ameliorate liver fibrosis in NAFLD by targeting the TGF-β/SMAD pathway [136]. In female Sprague Dawley rats fed with HFD, EGCG treatment (50 mg/kg, i.p. injection, 3 times per week, 8 weeks) was able to attenuate oxidative stress, steatosis, steatohepatitis, necrosis, and fibrosis in the liver through the NF-κB (limiting iNOS, COX-2, and TNF-α), TGF/SMAD (regulating matrix metalloproteinase-2/MMP-2, TIMP-2, and α-SMA), and PI3K/Akt/FoxO1 (relating to proliferation and trans-differentiation of HSCs) pathways [140]. A recent study also validated the anti-fibrotic effect of EGCG in NAFLD, by downregulating fibrosis-related genes COL I-α1, COL I-α2, COL III-α1, and COL IV-α3.

### 3.4. Prevention from HCC

At the late stage, NAFLD may develop into end-stage liver disease, i.e., cirrhosis, and eventually HCC, whereas HCC may occur regardless of the existence of cirrhosis. Oxidative stress, along with chronic and progressive inflammation, fibrosis, and cirrhosis, has been reported to significantly increase the risk of HCC development [141,142,143]. Effective approaches to treat NAFLD, with the aim of preventing or slowing down its progression into HCC, are urgently demanded to reduce the NAFLD-related mortality. EGCG might be a promising natural compound for chemoprevention of NAFLD-related liver tumorigenesis [144,145,146]. While the preventive effect of green tea and EGCG against tumorigenesis in NAFLD has been demonstrated in several animal models, the underlying mechanisms, especially causative links, have not been fully elucidated, thus further studies in this field are warranted to validate the effect with clear mechanisms of action.

In SHRSP.Z-Leprfa/IzmDmcr (SHRSP-ZF) rats, established by crossing stroke-prone spontaneously hypertensive rats with Zucker fatty rats, a NASH model was induced by HFD plus carbon tetrachloride injection (0.5 mL/kg BW, i.p., twice a week, 8 weeks), and administration of EGCG (0.1% in drinking water, 8 weeks) to the rats showed inhibitive effects on the development of preneoplastic HCC lesions, as revealed by the improved glutathione S-transferase placental form (GST-P)-positive foci by blocking renin-angiotensin system activation (serum angiotensin II, hepatic angiotensin-converting-enzyme and angiotensin II receptor 1 mRNA), decreasing oxidative stress (hepatic CYP2E1 and p-JNK proteins, and GPX and CAT mRNA), alleviating inflammation (serum TNF-α and IL-6, hepatic TNF-α, IL-6, IL-1β, and MCP-1 mRNA), and improving liver fibrosis (hepatic α-SMA protein, as well as the mRNA of α-SMA, procollagen-1, TGF-β1, MMP-2, MMP-9, TIMP-1, TIMP-2, and plasminogen activator inhibitor-1) [144]. In a NASH model in rats injected with a hepatic carcinogen diethylnitrosamine (DEN, 30 mg/kg BW, i.p., once) and fed with HFD, EGCG administration (0.01% and 0.1% in drinking water) could significantly inhibit the development of GST-P-positive foci (an indicator of preneoplastic HCC lesions), with the reduction in hepatic TG level, the improvements in hepatic oxidative stress (CAT and GPX), inflammation (TNF-α, IL-6, and IL-1β), and fibrosis (TIMP-1 and TIMP-2 mRNA), and the inhibition in excessive hepatocyte proliferation (cyclin D1 mRNA) [145]. While in male C57BL/6J mice, fed with HFD and injected with DEN, green tea extract (2% in diet) was observed to prevent the hepatic oncogenesis by inhibiting carcinogenic cascades related to NASH-related HCC, as indicated by the attenuated the frequency of proliferating cell nuclear antigen-positive hepatocytes, the decreased mRNA expressions of cyclin D1, MIB E3 ubiquitin protein ligase 1, oncostatin M, Ki-67, CD130, c-Fos, c-Myc, and survivin, and the increased apoptotic protease activating factor 1 mRNA [146].

In short, green tea and EGCG have shown potent effects on NAFLD in various animal and cellular models. The potential mechanisms of action may involve the improvements in oxidative stress, metabolism dysfunction, inflammation cascades, fibrotic response, and HCC tumorigenesis, in which the modulations in NRF2, AMPK, SIRT1, NF-κB, TLR4/MYD88, TGF-β/SMAD, and PI3K/Akt/FoxO1 signaling pathways are essential.

## 4. Beneficial Function of Green Tea and EGCG against NAFLD in Human Study

### 4.1. Clinical Trial

Tea is one of the most popular beverages all over the world, especially in eastern countries such as China, Japan, and Singapore. Drinking tea in a long term may benefit human health, e.g., reducing the risks of chronic diseases, including cancer, diabetes mellitus, cardiovascular diseases, neural diseases, and hepatic diseases [23]. It has been reported that consumption of green tea and its extract may benefit patients with NAFLD in clinical trials [147,148,149,150]. For example, in a trial with 38 NASH patients, treatment with tablet containing green tea extract (100 mg/tablet, 2 tablets/time, 3 times/day, 6 months) significantly improved body mass index (BMI), visceral fat to subcutaneous fat ratio, and liver to spleen ratio, as well as blood levels of glucose, lipids, alanine transaminase (ALT), aspartate transaminase (AST), and highly sensitive C-reactive protein (hs-CRP) [147]. In addition, a randomized placebo-controlled parallel-grouped trial involving 80 NAFLD patients showed that supplement with green tea extract capsule (500 mg/time, twice daily, 12 weeks) resulted in significant improvements in body weight, BMI, Homeostasis Model Assessment of Insulin resistance (HOMA-IR), lipid profiles (TC, TG, LDL-C, and HDL-C), inflammatory markers (hs-CRP, adiponectin), liver function indices (ALT, AST), and lipid accumulation in liver [148]. Moreover, in a randomized, double-blind placebo-controlled trial recruiting 67 NAFLD patients, intervention with green tea tablets (550 mg/time, once daily, 12 weeks) could also ameliorate some indices such as BMI, AST, and FBG, though not change body weight, ALT, HOMA-IR, ferritin, or total iron binding capacity [149]. Interestingly, in a randomized double-blind placebo-controlled study including 17 NAFLD patients, patients treated with a green tea beverage containing high-density catechins (1080 mg/700 mL, 700 mL/day, 12 weeks) were detected with significantly decreased body fat content, liver to spleen ratio, serum ALT level, and urinary 8-isoprostane excretion compared to those treated green tea containing low-density catechins (200 mg/700 mL, 700 mL/day, 12 weeks) and placebo (0 mg/700 mL, 700 mL/day, 12 weeks) [150]. These results further validate that catechins are the main bioactive components of green tea. Moreover, some certain positive results about the efficacy and safety of green tea and catechins for the management of NAFLD have been observed, indicating that it is worth recommending green tea and EGCG to the public with this regard. More clinical trials that are appropriately designed and conducted are warranted to confirm the protective effect of green tea and catechins in treating and managing NAFLD.

### 4.2. Systematic Review and Meta-Analysis

Systematic review and meta-analysis have been regarded as the most important approach for evidence-based medicine, which could contrast results from different studies, identify the pattern and source of disagreement among study outcomes, and reveal some interesting correlations under the condition of multiple studies. Through the aggregation of pooled information, a higher statistical power and more robust point estimate can be acquired by meta-analysis compared with any individual studies.

Several systematic reviews and meta-analyses have been conducted to assess the effect of green tea and tea catechin against NAFLD, providing further evidence that may remedy those shortcomings in an individual study. In a systematic review conducted in 2018, meta-analysis of four clinical trials comprising 234 subjects showed that supplementation of green tea or tea catechins significantly improved BMI (−2.08 (−2.81, −1.36) kg/cm^2^), ALT (−12.81 (−18.17, −7.45) U/L), AST (−10.91 (−19.66, −2.17) U/L), TG (−31.87 (−40.62, −23.12) mg/dL), TC (−27.57 (−36.17, −18.98) mg/dL), and LDL−C (−14.15 (−23.69, −4.60) mg/dL), though no significant effects were observed on HDL−C (7.41 (−1.49, 16.30) mg/dL) and HOMA−IR (−4.06 (−10.22, 2.09)) (presented as Mean Difference (95% confidence intervals), MD (95% CI)) [151]. This systematic review and meta-analysis suggested that green tea/tea catechin supplementation has potential benefits against NAFLD. However, in another systematic review and meta-analysis implemented in 2020 with 15 clinical trials, consumption of green tea, catechin-enriched green tea, or EGCG overall exerted no significant effect on liver enzymes such as ALT (−0.17 (−0.42 to 0.08)), AST (0.07 (−0.43, 0.29)), and ALP (−0.17 (−0.45, 0.10)) (expressed as Standardized Mean Difference (95% confidence intervals), SMD (95% CI)), whereas subgroup analyses disclosed that the interventions decreased the levels of liver enzymes in participants with NAFLD while increasing liver enzymes in healthy subjects [152]. The results of this study indicated that the effect of green tea/catechins/EGCG on liver enzymes is dependent on the health status of individuals, presenting as a moderate reducing effect in patients with NAFLD versus a slight increasing effect in healthy subjects. Taken together, it can be concluded that the effects of green tea and its components on NAFLD are certain in NAFLD patients, but various in healthy individuals. This phenomenon should be taken into consideration when selecting subjects for assessing the effects of green tea and its components against NAFLD.

## 5. Discussion

NAFLD is a highly prevalent disease worldwide, causing great burden to human health and society. It is estimated that 20% of individuals have the risk of NAFLD, and 25% of NAFLD patients may develop with NASH, while 4% of NAFLD patients and 20% of NASH patients may develop cirrhosis which indicates significant risk of HCC, liver transplantation, and liver-specific mortality [153,154,155]. Obese (in particular central obesity), T2DM, metabolic syndrome, hypertriglyceridemia, dyslipidemia, hypertension, and advanced age, etc., have been identified as the risk factors of NAFLD, though it can also be diagnosed in persons without these factors, including lean people and young children [155,156,157]. Currently, there is no specific medication for NAFLD, but lifestyle shift to a healthy pattern may make sense, e.g., weight loss, aerobic exercise regimen, as well as restrictions in calory, fructose, and alcohol, whereas treatment strategies including bariatric surgery (for patients unable to achieve weight loss goal), vitamin E (for patients without diabetes), pioglitazone (for patients with/without diabetes), statin (for patients suspected with cardiovascular risks), and liver transplant (for patients clinically decompensated) are available for NAFLD patients [37]. Notably, a wide range of targets including improvements in oxidative stress, insulin resistance, lipotoxicity, inflammation, and fibrogenesis have been proposed for NAFLD management. In particular, natural antioxidants (just as vitamin E) can be a promising candidate.

Green tea consumption, along with vegetable and fruit consumption, is one of the most important resources of natural antioxidants for people. Among the six categories of teas (green, yellow, white, oolong, black, and dark teas), green tea rich in catechins possesses the highest antioxidant capacity, which is also remarkably higher than that of most vegetables, fruits, edible flowers, and medicinal herbs [14,15]. EGCG is the most abundant catechin in green tea, and it has exerted potent antioxidant activity in vitro and in vivo. Green tea and EGCG have been reported with favorable effect on NAFLD and NAFLD-associated comorbidities including obesity, T2DM, metabolic syndrome, and cardiovascular diseases [19,20,24,25,26,27]. As evaluated in existing RCTs, administration with green tea extract and EGCG-enriched supplements is beneficial to patients with NAFLD/NASH, as per the improved indices and biomarkers related to liver function and histopathological alteration [147,148,149,150]. Further assessments by systematic review and meta-analysis have increased the evidence strength to a certain extent [151,152]. However, these RCTs are only in phase I or phase II, thus may not provide evidence that is strong enough to draw firm conclusions. We should also remind of the BIMBE (But It Might Be Endogenous) pitfall that may influence the reliability of such research. The patients with NAFLD and related risk factors including obesity, diabetes, and metabolic syndrome are very likely to pursue a healthy lifestyle by increasing exercise, reducing body weight, and decreasing calory intake. All these alterations are beneficial for NAFLD management. Randomized grouping, placebo control, and blind method are crucial strategies to appraise the effects of green tea and EGCG on NAFLD in comparison to those caused by lifestyle change. Still, though green tea consumption exerted a very limited side-effect, large doses of EGCG might cause mild acute adverse effects, including gastrointestinal disturbances (nausea and stomach injuries), especially consumed on an empty stomach. Special attention should be paid to the potential negative effects of EGCG in clinical trials. In a word, higher level clinical trials with good quality regarding rational methodology, multi-central cooperation, accurate data, and reliable conclusions are warranted to confirm the clinical efficacy and safety.

Besides EGCG, other catechins, including catechin and epicatechin, have also been shown to benefit NAFLD, which seems worthy of further exploration and application [158,159]. However, considering the contents of various catechins in dry green tea leaves (approximately, catechin: 1–3 mg/g, epicatechin 5–8 mg/g, and EGCG: 40–50 mg/g), it may be reasonable to attribute EGCG as the main bioactive compound responsible for the protective effect of green tea against NAFLD [14,15]. Thus, EGCG attracts the most attentions and is more widely investigated than other types of catechins. As compared with other antioxidants such as vitamin E, EGCG may possess some advantages, given that both EGCG and vitamin E have exerted considerable effects in NAFLD patients. Firstly, EGCG is rich in green tea, and may serve as the most important source of natural antioxidants, as tea beverage consumption has been regarded as the second largest worldwide (the first is water). In contrast, vitamin E is mainly from plant oils, vegetables, and fruits, but the contents are relatively low. It is easy to promote EGCG intake by increasing green tea consumption. In addition, EGCG is water-soluble, and may be transported through circulation system to different organs and excreted easily through urine, leading to the low possibility of side-effect by accumulation in the targets. However, vitamin E is fat-soluble, and may easily distribute and accumulate in tissues with rich lipids, inducing the increased risk of toxic effect. It has been reported that vitamin E supplementation may increase the risk of hemorrhagic stroke and prostate cancer, with conflicting reports of increased overall mortality in patients [37]. Promisingly, consumption of green tea and EGCG is only observed with very slight side-effects, which are usually tolerable to the patients, indicating a considerable safety.

Although in the recent 10 years, knowledge about the pathogenesis of NAFLD has increased dramatically with the occurrence of the “Two Hits Hypothesis” and “Multiple Parallel Hits Hypothesis”, it is still not fully clear and definite how NAFLD is originated and progressed. According to the updated literature, oxidative stress may play a central role in the development of NAFLD, through interacting with and accelerating the progression stages from single fatty liver to steatohepatitis, fibrosis, cirrhosis, and finally HCC. Notably, though the above sequence of events has been indicated in the general progression of NAFLD, it is not easy to separate them into completely independent stages, thus not easy to compare at which stage EGCG may be the most effective. However, considering that oxidative stress is an accelerator and promotor of inflammation, fibrosis, and even HCC, treatment with EGCG as an antioxidant to ameliorate oxidative stress may have the potential to block the progression of NAFLD. It may be expected that intervention with EGCG at an early stage is more rational for NAFLD management, as prevention is generally superior to curation for chronic diseases. Daily consumption of green tea or supplementation of EGCG deserve recommendation for individuals who are suspected with the risk of NAFLD or NAFLD-related complications, to prevent or slow down the occurrence of end-stage liver diseases, whereas further clinical trials and evidence-based medicine data are warranted to carry out this health promotion strategy to the public.

As for the underlying mechanisms involved in the hepato-protective effects green tea and EGCG against NAFLD, a wealth of studies has pointed to the amelioration in oxidative stress, accompanied with inhibitions in metabolism dysfunction, inflammatory cascades, fibrogenic response, and tumor initiation [23]. Signaling pathways, including NRF2, AMPK, SIRT1, NF-κB, and TGF-β, are highly implicated in the mechanisms of action. In the future, plenty of mechanism studies that may establish causative links in the pathological processes of NAFLD, and put forward the treatment strategies, are still warranted in this regard. Potential directions include, but are not limited to, regulations in apoptosis, autophagy, immune, and epigenetics/epigenomics.

## 6. Conclusions

In conclusion, oxidative stress may play a central role in the development of NAFLD. Oxidative stress induced by excessive ROS triggers lipid peroxidation and release of inflammatory molecules, which promote hepatic lipid accumulation, inflammation, fibrosis, cirrhosis, and eventually HCC. Green tea and its most abundant catechin EGCG have been reported with beneficial effects against NAFLD in RCTs, systematic reviews, meta-analysis, and animal studies. The mechanisms may include, not only alleviating oxidative stress, but also improving lipid metabolism, inflammation cascades, fibrotic response, and HCC tumorigenesis, in which the regulations in the NRF2, AMPK, SIRT1, NF-κB, TLR4/MYD88, TGF-β/SMAD, and PI3K/Akt/FoxO1 pathways are highlighted. Further studies are warranted to validate the effect of green tea and EGCG on NAFLD, especially the end-stage liver diseases such as HCC, with clear illustration on the underlying mechanisms of action.
